# Social networks as predictors of colorectal cancer screening in African Americans

**DOI:** 10.21633/jgpha.6.306

**Published:** 2017

**Authors:** Ernest Alema-Mensah, Selina A. Smith, Mechelle Claridy, Victor Ede, Benjamin Ansa, Daniel S. Blumenthal

**Affiliations:** 1 Department of Community Health and Preventive Medicine, Morehouse School of Medicine, Atlanta, GA; 2 Cancer Research Program, Morehouse School of Medicine, Atlanta, GA; 3 Department of Family Medicine, Medical College of Georgia, Augusta University, Augusta, GA; 4 Satcher Health Leadership Institute, Division of Behavioral Health, Morehouse School of Medicine, Atlanta, GA; 5 Institute of Public and Preventive Health, Augusta University, Augusta, GA

**Keywords:** Colorectal cancer screening, social networks, African Americans

## Abstract

**Background:**

Early detection can reduce colorectal cancer (CRC) mortality by 15%–33%, and screening is widely recommended for average-risk adults beginning at age 50 years. Colorectal cancer mortality rates are higher in African Americans than in whites, while screening rates are somewhat lower. Individual social networks can reduce emotional and/or logistical barriers to health-promoting but distasteful procedures such as CRC screening. The aim of this study was to examine social network interactions, and their impact on CRC screening among African Americans. We hypothesized a positive association between social network index (SNI) scores and CRC screening.

**Methods:**

In a community intervention trial with four arms, we previously demonstrated the efficacy of a small group educational intervention to promote CRC screening among African Americans. This intervention outperformed a one-on-one educational intervention, a reduced out-of-pocket expense intervention, and a control condition. In the present analysis, we compared the SNI scores for participants in the small group intervention cohort with a comparison group comprised of the other three cohorts. Social networks were assessed using the Social Network Index developed by Cohen.

**Results:**

Small group participants had a significantly higher network diversity score (Mean difference 0.71; 95% CI, 0.12–1.31; p=0.0017) than the comparison group. In the second component of the SNI score – the number of people talked to over a two week period – the small group intervention cohort also scored significantly higher than the comparison group. (Mean difference, 9.29; 95% CI, 3.963–14.6266; p=0.0004).

**Conclusions:**

The findings suggest that social interaction and support was at least partially responsible for the relatively high post-intervention screening rate in the small group intervention participants. Education in small groups could foster strong social networks. Strong and positive network diversity and a large number of people in social networks may enhance CRC screening rates among African Americans.

## INTRODUCTION

Colorectal cancer (CRC) is the third most common cause of cancer-related death in the United States, and the estimated new cases and deaths from CRC in 2016 are 134,490 and 49,190 respectively (National Cancer Institute, SEER 2009–2013 data). Early detection can reduce CRC mortality by 15%–33%, (Burch et al., 2007) and screening is widely recommended for average-risk adults beginning at age 50 years (U.S. Preventive Services Task Force (USPSTF), 2008).

Data from the 2014 Behavioral Risk Factor Surveillance System showed that only 66.6% of US adults aged 50–75 years have fully met the USPSTF recommendation for CRC screening (Centers for Disease Control and Prevention [CDC], 2015). Factors associated with higher screening rates include higher income, higher education, older age, and male sex; strong social ties and supportive relationships; better health care provider communication; and a physician’s recommendation for testing (Tessaro et al., 2006). Individual social networks reduce emotional and/or logistical barriers to CRC screening participation (Manne et al., 2012; Schoenberg et al., 2016). In particular, social support is related to CRC screening adherence among African Americans (Kinney et al., 2005; Brittain et al., 2012).

African Americans, compared to whites, have disproportionately higher incidence and mortality rates and lower screening rates for CRC. An estimated 17,240 cases and 7,030 deaths of CRC are expected to occur among blacks in 2016. Incidence rates in black males and females compared to whites are 27% and 22% higher, respectively.

Mortality rates for CRC are 52% higher in black men and 41% higher in black women compared to white men and women (American Cancer Society, 2016). It is estimated that 19% of the racial disparity in CRC mortality rates can be attributed to lower screening rates and 36% to lower stage-specific survival among blacks (Lansdorp-Vogelaar et al., 2012). CRC screening rates are slightly lower among blacks compared to whites, 59% versus 61%, respectively (CDC, 2015).

In a previous study known as the Colorectal Cancer Screening Intervention Trial (CCSIT) (Blumenthal et al, 2010), we demonstrated the efficacy of a small group educational intervention in promoting CRC screening among African Americans. In that project, African-American men and women aged 50 years (N=312) and above were enrolled in a randomized, controlled community intervention trial. We compared post-intervention screening rates among participants in a cohort receiving the small group intervention with participants in cohorts receiving a one-on-one educational intervention, a reduced out-of-pocket expense intervention, and no specific intervention (control group) except for some printed material. The small group intervention was the only one that out-performed the control group at a statistically significant level.

As noted above, socioeconomic and psychosocial factors are associated with adherence to USPSTF recommendations for CRC screening. In the current report, we further analyze the results of the previous study to compare the social interaction networks of the small group intervention cohort with the networks of the other 3 cohorts, using a validated Social Network Index (SNI). We hypothesized a positive association between SNI scores and CRC screening.

## METHODS

Social networks were assessed using the Social Network Index (SNI) developed by Cohen (1991). Cohen et al describe the SNI as follows (Cohen et al, 1997):

The Social Network Index assesses participation in 12 types of social relationships. These include relationships with a spouse, parents, parents-in- law, children, other close family members, close neighbors, friends, workmates, schoolmates, fellow volunteers (e.g., charity or community work), members of groups without religious affiliations (e.g., social, recreational, or professional), and members of religious groups. One point is assigned for each type of relationship (possible score of 12) for which respondents indicate that they speak (in person or on the phone) to someone in that relationship at least once every 2 weeks. The total number of persons with whom they speak at least once every 2 weeks (number of network members) is also assessed.

Cronbach’s alpha for the SNI scale was 0.52 in this study. Descriptive statistics and bivariate analyses were run. T-test statistics and logistic regression analysis were used to determine differences between groups. SAS 9.4 software (SAS Institute. 2013) was used for all analyses.

## RESULTS

The sociodemographic characteristics of the study participants by screening status is illustrated in Table 1. There were a total of 312 participants, mostly females (71%, n=224), between 50 and 64.9 years (40.9%, n=131), with a high/technical school education (45.7%, n=142) and were not married (71.7%, n=223).

For the purposes of this analysis, scores for the Small Group Education cohort were compared with those of a comparison cohort comprised of the other participants in the community intervention trial: those in the Reduced Out-of-Pocket Expense, One-on-One Education, and Control cohorts. Table 2 shows the results of the bivariate analyses performed for the study. There was a statistically significant difference in the Network Diversity score and the Total number of People Talked to/2 weeks. Small group participants had a higher network diversity score (Mean difference 0.19; 95% CI, −0.35–1.72; p=0.0042) compared to the comparison group. Total Number of People Talked to/2 weeks was also significantly higher among the small group participants than the comparison group (Mean difference, 6.30; 95% CI, 0.47–12.12; p=0.001).

Table 3 shows the result of the multivariate modelling using logistic regression. None of the potential confounders was explanatory.

## DISCUSSION

In the original CCSIT study, participants in a small group educational intervention were more likely to be screened subsequently for colorectal cancer than were persons who received one-on-one education with a health educator, persons who were offered reimbursement for out-of-pocket expenses associated with CDC screening, and persons who did not participate in any intervention but received some printed educational material. This led to the research question: what was the characteristic or quality of an educational experience in a small group that motivated participants to pursue CRC screening? We based our explanatory hypothesis on social support theories, such as Social Ecological Theory (Breslow, 1996) and Social Cognitive Theory (Bandura, 1986). These theories suggest that social and emotional support received from others in the small group (and elsewhere) may have encouraged participants to overcome psychological and other barriers to screening. We tested our hypothesis by utilizing the Social Network Index developed by Cohen (1991) who employed it to demonstrate that individuals with more diverse social ties are less susceptible to upper respiratory infections (Cohen et al, 1997). In our study, we found that such persons were more likely to pursue screening for colorectal cancer. Colorectal cancer screening is viewed with distaste even by many people who are quite aware of the disease and the fact that screening can detect it early or even prevent it altogether. Neither of the two most common screening methods – colonoscopy and fecal occult blood testing – is appealing. But it appears that the encouragement and support of others in an individual’s social network can help counteract the distaste for screening.

## CONCLUSIONS

This analysis provided evidence to confirm our hypothesis that participants who experienced the small group educational intervention – and subsequently had a higher CRC screening rate than members of the other three cohorts – would have a higher overall SNI score. This suggests that social interaction and support is at least partially responsible for the relatively high post-intervention screening rate in the small group intervention participants.

## Figures and Tables

**Table 1:** Sociodemographic characteristics of study participants by screening status at follow-up

Characteristics	No CRC Screening n (%)	CRC Screening n (%)	Chi-square	p-value
**Sex**			1.1006	0.2941
Male	79 (89.8)	9 (10.2)		
Female	191 (85.3)	33 (14.7)		
**Age**			3.5862	0.0583
50–64.9 years	119 (90.8)	12 (9.2)		
65+ years	151 (83.4)	30 (16.6)		
**Education**			1.5855	0.4526
Elementary	51 (82.2)	11 (7.7)		
High/Technical school	126 (88.7)	16 (11.3)		
College	92 (85.9)	15 (14.0)		
**Marital status**			0.1689	0.6811
Married	75 (85.2)	13 (14.8)		
Other	194 (87.0)	29 (13.0)		
**Insurance coverage**			0.0134	0.3502
No insurance	23 (95.8)	1 (4.2)		
Medicare/Medicaid	174 (84.9)	31 (15.1)		
Health insurance/HMO	73 (87.9)	10 (12.0)		

**Table 2:** Bivariate analysis for Social Network Index

Variables	Mean	95% Confidence Interval		p- value
		Lower	Upper	
**Network diversity**				
Small group score (n=68)	5.809	5.437	6.181	
Comparison group score (n=244)	5.623	5.361	5.885	0.0042
**Total number of people talked to/2 weeks**				
Small group (n= 68)	32.618	26.111	39.125	
Comparison Group (n=244)	26.320	23.319	28.827	0.001

**Table 3:** Logistic regression

		95%Confidence Interval		p-value
Variables	OR	Lower	Upper	
**Gender**				
Male vs Female	1.524	0.638	3.639	0.3425
**Insurance**				
Medicaid/Medicare vs No insurance	2.77	0.297	25.802	0.3709
Health insurance/HMO vs No insurance	2.287	0.244	21.458	0.469
**Age**				
65+ vs 50–64.9 years	2.102	0.741	5.963	0.1626
**Education**				
High/Technical school vs Elementary school	0.405	0.143	1.144	0.088
College/Graduate school vs Elementary school	0.42	0.137	1.285	0.1283
**Marital status**				
Other vs Married	0.923	0.375	2.274	0.8619
**Social Network Index**				
Network diversity score	1.246	0.9	1.724	0.1851
Number of people talked to/2 weeks	0.953	0.899	1.011	0.1123
